# Let’s talk about genes, and I dont mean trousers: encouraging cancer genetics literacy amongst children

**DOI:** 10.3332/ecancer.2014.408

**Published:** 2014-02-27

**Authors:** Rachel Iredale, Kim Madden

**Affiliations:** Genomics Policy Unit, University of South Wales, Pontypridd, CF37 1DL, UK

**Keywords:** cancer genetics, children, ethics, health literacy, public engagement, family history

## Abstract

Acquiring genetic literacy is one of the most important things a person can do to promote their own and their family’s health. Family history—genetics and the shared environment—is a significant risk factor for cancer as well as other common diseases, such as cardiovascular disease and diabetes. A good understanding of family health history should increasingly be used to personalise health messages and promote healthy lifestyles. The Let’s Talk About Genes project explored whether it was feasible and acceptable to engage young children in Wales with family history as it relates specifically to cancer, so they increase their cancer genetics literacy over time and become more aware of general health issues that relate to cancer.

## Introduction

A recent *ecancermedicalscience* editorial discussed Angelina Jolie and cancer genetic decision-making [7]. It might be argued that Angelina Jolie has done more for cancer genetics in the last few months than the last 15 years of public engagement activity. Those of us working in the field of cancer genetics, particularly in relation to the public understanding of genetics, can be in no doubt that acquiring genetic literacy is one of the most important things a person can do to promote their own and their family’s health. Family history—genetics and the shared environment—is a significant risk factor for cancer as well as other common diseases, such as cardiovascular disease and diabetes. We would argue that a good understanding of family health history should increasingly be used to personalise health messages and promote healthy lifestyles.

As early as 1996, young peoples’ understanding of family history and their perceived vulnerability to common conditions was being explored [8]. Yet, recently, a report of a meeting held by the National Human Genome Research Institute asked what it means to be genomically literate, and examined the challenge of achieving genomic literacy, for the general public from kindergarten right through to adult education [4]. With the Genetics and Merthyr Youth (GAMY) Project, we worked with a group of teenagers 16–19 years of age in a deprived South Wales valley town over a period of 18 months and discovered that young people’s attitudes to genetics are complex and not easily generalisable [5]. There were low levels of familiarity with, and knowledge of, genetics from the outset. Most of the young people did not have pre-existing attitudes towards genetics and had been given little or no thought to the topic before the project began. However, levels of awareness and general genetic literacy increased as the project progressed, and we realised that over time young people can develop an awareness of genetics that makes sense to them; they demonstrate that they can think creatively about genetics, and they are able to engage in considering genetic and other risk factors when thinking about health and disease.

Therefore, in the Let’s Talk About Genes project, we wanted to explore whether it was feasible, and whether it was acceptable, to engage much younger children in Wales with family history as it relates specifically to cancer, so they increase their cancer genetics literacy over time and become more aware of general health issues that relate to cancer. We proposed spending three days with a group of up to 20 children in a school, using fun activities, games, and creative workshops to explore issues around genetics and cancer and risk. We discovered that it was certainly feasible to do this—with many primary schools keen on participating right from the outset—but that it was simply unacceptable to many people to engage any person under the age of 16 years with the notion that cancer—or any other illness—might be inherited or might run in families.

## Ethical considerations

In the beginning, we wanted to approach primary school children aged seven to eight years old, but the funding body was opposed to this. The funders had sent the project proposal for peer review, and whilst the reviewers agreed to the funding, they were nervous about the proposed age of the participants. They suggested that we raise the age limit to children who were 10–11 years old, i.e., those who were in their last year of primary school education in the United Kingdom. The funders also required us to submit the project to the NHS National Research Ethics Service (www.nres.nhs.uk). Before submitting the paperwork to the ethics committee, we had agreement from two local primary schools that they would like to take part in the project. Unfortunately, when we presented the project in person at the committee, they rejected the notion that children of this age would be able to discuss cancer and genetics, saying ‘linking the genetic with the familial may cause undue worry and distress in the participants’ and that committee ‘members ...were not convinced that children of this age would be able to interpret that information correctly’. This was despite the fact that two head teachers and their schools’ Board of Governors had confirmed that the 10- and 11-year-old children in their schools would be able to understand the genetics information that we proposed to discuss. The ethics committee advised us to conduct the project with even older children and invited us to resubmit.

We secured the agreement of a local mixed secondary school with written support from the school’s senior management team, including the Head of Science, to carry out the project with their children. After more paperwork than you can imagine and two more fraught personal appearances in front of the ethics committee, we were finally granted permission to work with children aged 12–13 years old. In the United Kingdom, 12- and 13-year-old girls are routinely offered the HPV vaccine to protect against cervical cancer, and this typically takes place in their second year of secondary school. We felt that children of this age would, and should, be able to discuss cancer, and genetics. It took 16 months from the proposal submission to the final approval for a three-day project.

However, a number of conditions were placed upon us before we could proceed: (1) we had to hold information sessions for parents at the school. (2) Consent had to be obtained separately from parents and children. (3) We had to write up a detailed lesson plan outlining every activity that would be undertaken. (4) We had to establish an independent monitoring group to oversee the project, which was to include parents, and the ethics committee had to see a list of members of this group; had to see exactly what they would be sent and details had to be provided about how often they would meet. (5) Adverse events had to be defined in advance, and details of stopping rules agreed. (6) There had to be a named person at the school and in the local genetics centre who could offer support to the children and parents as necessary. (7) A genetic counsellor had to be present in the classroom at every session.

One might query who the real ‘experts’ are in research of this nature. The funding body is a leading cancer charity in Wales. They were cautious about us working with such a young age group, nevertheless they also acknowledged the potential benefit of such a project and the importance of the research proposed, by suggesting a slightly older age group, indicating that it is acceptable and appropriate. However, although the funders agreed in principle to the funding, they also absolved themselves of any responsibility by transferring the decision making and final approvals over to an NHS ethics committee.

Similarly, in reviewing our application, the ethics committee focused on the complexity of linking genetics and cancer, and their discussions were driven by their own experiences and encounters with the topic. They highlighted concerns about the potential research burden for participants and the capacity of children to make sense of such information. The agenda pursued by the ethics committee overshadowed discussion of other factors relevant to the review process, such as the research design and methods. It is right and reassuring that committees explore these issues as they work to safeguard participants in research that is perceived to be emotive or challenging. However, the approach and process diminished the view of other experts, particularly the teaching professionals who were in everyday contact with these children.

It is clear that the way in which the funders and ethics committee conceptualised the research activity shaped their responses and reaction to the proposed study. From the perspective of schools and teachers, they felt that what was being proposed was complementary to the activities with which children are involved in their schools. They did recognise potentially sensitive issues that may arise as a result of children learning about cancer and genetics; however, they felt that this was no more ‘concerning’ than other school activities. This raises some challenges for any future work with children in this area—how do we reassure committees, and how do we get them to acknowledge the extent of their expertise alongside other relevant experts?

## Children and cancer genetics

The Let’s Talk About Genes project tried to demonstrate whether it was feasible and acceptable to engage children with family history as it relates to cancer, so they increase their cancer genetics literacy over time, and become more aware of general health issues that relate to cancer. A total of 320 participant information packs were sent out to parents, with 65 sets of consent forms returned (separate forms from both parents and children). Twenty children were randomly selected—ten boys and ten girls—and took part in activities and workshops during their normal school day over three days. We explored:

- How aware are children of genetics?
- How aware are children of cancer?
- Whether children can explore the role that family history plays in cancer.
- Whether children can learn about healthy behaviours that are likely to reduce cancer risk.
- Whether children can act as disseminators of cancer genetics information to their peers.

For each workshop, a detailed timetable of activity was developed, discussed, and reviewed by the research team. Learning aims and objectives were identified alongside methods for collecting data. Within the programme of activity we were able to explore biological inheritance as well as familial experiences in terms of shared environments and behaviours. We explored, through the use of games, how our risk of developing cancer is affected by some factors that we can control (e.g., diet and lifestyle) and some that we cannot (our genes). Although the focus of the study introduced participants to genetics and family history as it relates to cancer, this was situated within a broader context of cancer and health-promotion messages relevant to the disease as a whole.

Baseline and exit data were obtained via handheld electronic voting tools and explored the children’s views on health, inheritance, genetics, and cancer. The children engaged in a variety of activities and discussions over the three days, which revolved around these four discreet topics. In one activity, the children were divided into groups and explored factors associated with health by recording their ideas on a body stencil, which they obtained through drawing around a member of their group. This activity was used to capture participants’ first impressions and understanding of health before any genetics and cancer learning. In exploring ideas and notions of inheritance, participants completed group collages and individual portraits, as well as took part in a large group survey of traits. There was an opportunity to raise anonymous questions with a cancer expert during a question and answer (Q & A) session, and there was a group discussion with a consultant in clinical cancer genetics. We accessed and adapted learning resources from http://learn.genetics.utah.edu/ and devised additional games to help explain and explore notions of risk and chance as they related to genetics and cancer. We employed the skills of a graphic note-taker to record key comments and observations from all group activities and discussions, providing a reference map and point of reflection for the participants and research team alike.

The participant-led approach to the group discussions and, in particular, the Q & A with a cancer expert was highly valued amongst the participants. As one young person remarked, ‘Magazine cutting into collages for family history was fun, and I would be happy to do it again. Also, I loved drawing on people. I also loved the question and answers session’ as all of my questions were answered thoroughly and I don’t have any questions that weren’t answered’. Kinaesthetic and creative activities were also well received by participants and resulted in the production of a number of creative outputs as well as the final rap song.

At the end of the three days, the children came up with an animated rap, which was agreed as being something useful that their peers could use to convey some initial ideas about cancer and genetics. We discovered that children do have some awareness about cancer, but lower levels of awareness around genetics. They are able to recognise that some traits are genetically inherited whilst others are a result of their environment. They are able to recognise key health-promotion messages about cancer, risk, and lifestyle, with one participant saying ‘The one thing that has stuck in my mind is that you can reduce your risk of cancer massively’. Finally, we learned that engaged children are able to communicate with confidence; log onto and listen to their cancer genetics rap *Let’s Talk About Genes and I Don’t Mean Trousers* and judge for yourself—(Video 1).

**Tap your feet in a 4/4 beat and rap about genetics**
*Let’s talk about genes/And I don’t mean trousers/WOWZERS!*
*It’s a lot to take in/From Stanwell’s pupils/Let’s begin.*
*Cancer is something that affects 1 in 3/Many different types/Diet and genes.*
*It also can contribute/To you getting ill/But a healthy life choice/Helps a strong will.*
*You should always remember/To look after yourself/Stay fit/You can’t put a price on your health.*
*Always check yourself/Be body aware/Make sure you check/Under here and over there.*
*In a lifetime there can be many causes/Drinking and smoking/Even the sun can cause it.*
* Be careful of the summer/Hat and sun cream/Keep protected with Factor 50.*
*A tan is visible proof/That your skin is damaged/If you lay off the sun bed/It can be managed.*
*Another effect is family history/Cancer running in the family/Can be a mystery.*
*Small, tall, fat and thin/How you look comes from within/GENETICS!*
*Knowing your family history/Is important/To know if you have the genes/Are they dormant?*
*Chromosome is a DNA strand/And it’s the stuff/That makes your hand.*
*Genotype is a recipe of you/Passed down from your ancestors to you.*
*Genetics is a study of inheritance/To learn about history/Could be relevant.*
*All humans have the same genes/So you’re the same as me/It’s a basic recipe.*
*Patterns of inheritance/Are hard to predict/Did you know/Only 5% are genetic.*
*An equal number of traits/Are passed down/From parents to children/The circle turning around.*
*In case you didn’t know/Cancer can be fatal/Even your pets/Could feel the effects.*
*There are about 200 different cancers/You can use the internet/To find some answers.*
*It’s definitely not cool/And I’m not joking/Cancer risk is heightened/If you’re smoking.*
*Chemotherapy can make you lose your hair/Or you can wear a cap/To stop that.*
*40% of cancers are preventable/Take a look at how you live/Keep it sensible.*
*A healthy diet and exercise/Will stop you going up a size.*
*Thanks to medicine/Opening doors/Now oncologists/Are working on cures.*
*Let’s talk about genes/And I don’t mean trousers/WOWZERS!*
*It’s a lot to take in/Stanwell’s pupils gonna tell you something.*
*GENETICS!*

Genomic discoveries will bring numerous opportunities for improving human health. Key to these potential improvements is public understanding and acceptance of these new developments [2]. Background knowledge of family cancer history is essential for estimating an individual’s cancer risk and making clinical recommendations regarding screening or referral to specialist cancer genetics centres. The earlier you know which conditions run in your family, the better your chances of adopting preventative measures. Similarly, we need to improve our preparedness to integrate genomic technologies into our healthcare systems to truly personalise medicine in the future. Since 2004, in the United States, Thanksgiving Day has been declared National Family History Day by the Surgeon General (https://familyhistory.hhs.gov/fhh-web/home.action).

Yet, we still know very little about young people’s attitudes to cancer genetics. Family health history tools are widely available, and it has been argued by Ashida et al [1] that older individuals need to be empowered to provide social resources within familial networks where susceptibility to inherited cancer is suspected [3]. The Let’s Talk About Genes project showed us that anti-genetics attitudes still prevail in society. Far from making genetics something to be discussed easily and naturally, we risked making children afraid of the topic, with counsellors and teachers lurking on the sidelines and the possibility of an independent monitoring group popping into the classroom at any time. Without replicating the entire approval process, it is unclear whether NHS ethics committees in Wales are particularly risk averse and are more anti-genetics than anywhere else. There is a well established All-Wales Medical Genetics Service, and our research unit has spent over 15 years exploring the impact of genetics on Welsh society. However, unless we encourage health-care professionals, teachers, and others to facilitate genetic conversations with our children, there is no chance that genetics literacy will improve for this generation. Some have argued that the genotyping of children as part of personalised screening programmes for common cancers may become commonplace. In the meantime though, might the Angelina Jolie effect and the culture of celebrity make it more acceptable instead for us to talk about cancer and genetics with our kids in the future?

## Conclusion

The Let’s Talk About Genes project was a feasibility study that explored the acceptability of discussing cancer and genetics with school children in Wales. We conclude that it is both feasible and acceptable to engage children with family history as it relates to cancer, so they increase their cancer genetics literacy over time, and become more aware of general health issues that relate to cancer. Children are increasingly exposed to both cancer and genetic concepts. What is of utmost importance as we move forward with public health initiatives in cancer genetics is that children and young people are engaged in a developmentally appropriate manner. Those responsible for driving forward these initiatives must be better informed about the developmental trajectory of children into adulthood as well as children’s ability to deal with such issues, and they must be guided by experts involved with children on a day-to-day basis.

## Figures and Tables

**video 1.**
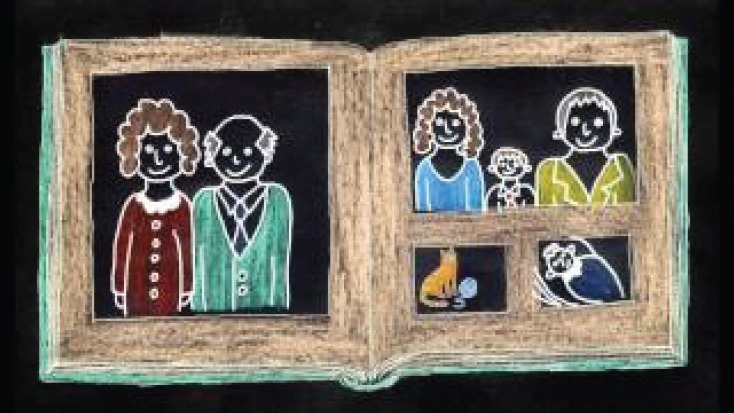
The cancer rap. To view this video, click here:www.youtube.com/genomicspolicyunit.
